# Multimodal assessment improves neuroprognosis performance in clinically unresponsive critical-care patients with brain injury

**DOI:** 10.1038/s41591-024-03019-1

**Published:** 2024-05-30

**Authors:** B. Rohaut, C. Calligaris, B. Hermann, P. Perez, F. Faugeras, F. Raimondo, J-.R. King, D. Engemann, C. Marois, L. Le Guennec, L. Di Meglio, A. Sangaré, E. Munoz Musat, M. Valente, A. Ben Salah, A. Demertzi, L. Belloli, D. Manasova, L. Jodaitis, M. O. Habert, V. Lambrecq, N. Pyatigorskaya, D. Galanaud, L. Puybasset, N. Weiss, S. Demeret, F. X. Lejeune, J. D. Sitt, L. Naccache

**Affiliations:** 1 Sorbonne Université, Paris, France; 2 Paris Brain Institute - ICM, Inserm, CNRS, PICNIC-Lab, Paris, France; 3 APHP, Hôpital de la Pitié Salpêtrière, DMU Neurosciences - Neuro ICU, Paris, France; 4 GHU Paris Psychiatrie et Neurosciences, Pole Neuro, Sainte‑Anne Hospital, Anesthesia and Intensive Care Department, Paris, France; 5 Laboratoire des systèmes perceptifs, Département d’études cognitives, École normale supérieure, PSL University, CNRS, Paris, France; 6 APHP, Hôpital de la Pitié Salpêtrière, DMU Neurosciences - Neurophysiology, Paris, France; 7 Physiology of Cognition GIGA-CRC In Vivo Imaging Center, University of Liège, Liège, Belgium; 8 APHP, Hôpital de la Pitié Salpêtrière, Departement of Nuclear Medicine, Laboratoire d’Imagerie Biomédicale, Inserm, CNRS, Paris, France; 9 APHP, Hôpital de la Pitié Salpêtrière, Departement of Neuro-radiology, Paris, France; 10 APHP, Hôpital de la Pitié Salpêtrière, Departement of Neuro-anaesthesiology and Neurocritical care, Paris, France; 11 Paris Brain Institute - ICM, Inserm, CNRS, Data Analysis Core, Paris, France

**Keywords:** Disorders of consciousness, Prognosis, Medical ethics, Prognostic markers

## Abstract

Accurately predicting functional outcomes for unresponsive patients with acute brain injury is a medical, scientific and ethical challenge. This prospective study assesses how a multimodal approach combining various numbers of behavioral, neuroimaging and electrophysiological markers affects the performance of outcome predictions. We analyzed data from 349 patients admitted to a tertiary neurointensive care unit between 2009 and 2021, categorizing prognoses as good, uncertain or poor, and compared these predictions with observed outcomes using the Glasgow Outcome Scale–Extended (GOS-E, levels ranging from 1 to 8, with higher levels indicating better outcomes). After excluding cases with life-sustaining therapy withdrawal to mitigate the self-fulfilling prophecy bias, our findings reveal that a good prognosis, compared with a poor or uncertain one, is associated with better one-year functional outcomes (common odds ratio (95% CI) for higher GOS-E: OR = 14.57 (5.70–40.32), *P* < 0.001; and 2.9 (1.56–5.45), *P* < 0.001, respectively). Moreover, increasing the number of assessment modalities decreased uncertainty (OR = 0.35 (0.21–0.59), *P* < 0.001) and improved prognostic accuracy (OR = 2.72 (1.18–6.47), *P* = 0.011). Our results underscore the value of multimodal assessment in refining neuroprognostic precision, thereby offering a robust foundation for clinical decision-making processes for acutely brain-injured patients. ClinicalTrials.gov registration: NCT04534777.

## Abstract (web summary)

Multimodal approaches combining various numbers of behavioral, neuroimaging and electrophysiological markers improves neuroprognosis performance in clinically unresponsive critical-care patients.

## Main

Prognostic evaluation of unresponsive patients following acute brain injury is one of the most difficult medical, scientific and ethical challenges. Indeed, withdrawal of life-sustaining therapies (WLST) is the leading cause of death in this setting, and these decisions are based on the prognosis and patients’ wishes^1–4^. Disease-specific scores and decision algorithms have been developed to help physicians reduce uncertainty when predicting functional outcomes, especially for common etiologies such as anoxia^5,6^ and traumatic brain injury (TBI)^7^. These decision aids, as well as both recent European and US guidelines on the assessment of patients with disorders of consciousness (DoC), recommend using a multimodal assessment (MMA) combining several metrics derived from behavioral, neuroimagery and electrophysiology when initial behavioral assessment is non-univocal or in the presence of confounding factors^4,8–11^.

Although the idea of improving neuroprognosis performance by increasing the amount of available evidence seems to be a rational approach, it has not been empirically validated in clinical practice. Moreover, multimodal approaches increase the odds of discrepancies across markers that could lead to choice paralysis or to biased decisions^12,13^.

In this paper, we aimed to evaluate the performance of MMA in determining the neuroprognosis of patients with acute brain injury. We report a 12-year analysis of MMA performance in patients assessed at a specialized French medical center, and we provide evidence that MMA indeed decreases uncertainty and improves the accuracy of long-term functional outcome predictions.

## Results

### Population description

**Fig. 1:**
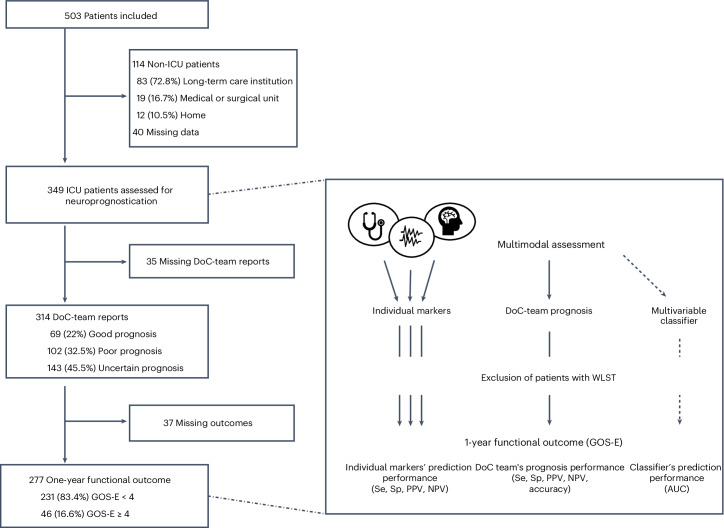
Flowchart and study design. Data-collection flowchart (left) and schematic illustrating study design (right). ICU, intensive care unit; DoC, disorder of consciousness; GOS-E, Glasgow Outcome Scale–Extended (levels range from 1 to 8, with higher levels indicating better outcomes); Se, sensitivity; Sp, specificity; PPV, positive predictive value; NPV, negative predictive value; AUC, area under the receiver operating characteristic (ROC) curve.

Of the 503 patients included in the cohort between January 2009 and August 2021, 349 intensive care unit (ICU) patients met our inclusion criteria, 114 (22.7%) were non-ICU patients and 40 (8%) had missing data. The vast majority (96%, *n* = 335) of the patients were referred from other ICUs for expert assessment of consciousness and neuroprognosis. The prognosis determined by the DoC team, a multidisciplinary group of neurointensivists, neurologists, neurophysiologists, neuroradiologists and neuroscientists, and one-year functional outcomes were available for 277 (79%) patients (Fig. 1).

**Extended Data Fig. 1:**
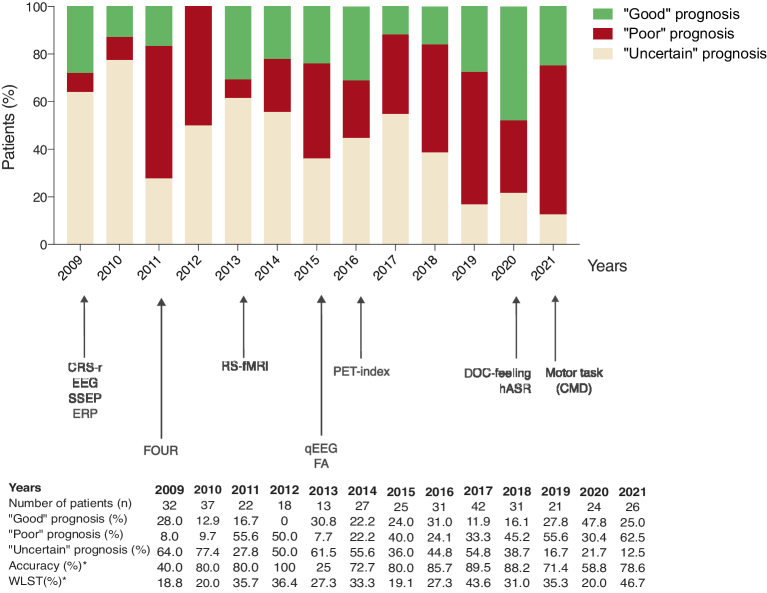
Distribution of DoC-team prognosis and implementation of individual markers over the study period. When split on the median ( ≥ or < 2016), there is a significant decrease in the proportion of “uncertain” prognosis with a relative increase of “poor” prognosis (54.84% vs 36.48%, OR = 0.47 [95%CI: 0.29 - 0.76], *P* = 0.001 and 25.81% vs 38.99%, OR = 1.83 [95%CI: 1.11 – 3.06], *P* = 0.016, respectively) with no difference in terms of “good” prognosis (19.35%, vs 24.53%, OR = 1.35 [95%CI: 0.76 - 2.41], *P* = 0.279), accuracy (73.21% vs 78.95%, OR = 1.37 [95%CI: 0.58 – 3.16], *P* = 0.431) and WLST (26.5% vs 34.51%, OR = 1.46 [95%CI: 0.83 - 2.60], *P* = 0.179). Two-sided Fisher’s exact test used with no adjustment for multiple comparisons. CRS-r: Coma Recovery Scale revised; (q)EEG: (quantitative) Electroencephalography; SSEP: Somatosensory Evoked potential; ERP: Event Related Potential (‘Local-Global’ paradigm); FA Fractional Anisotropy; FOUR: Full Outline of UnResponsiveness Score; RS-fMRI: Resting state – functional Magnetic Resonance Imaging; PET: Positron Emission Tomography; DoC: Disorder of Consciousness; hASR: habituation of Auditory Startle Reflex; CMD: Cognitive Motor Dissociation. *: for % Accuracy and % WLST the percentages are provided on n = 277 and 259, respectively.

Between 2009 and 2021, the median number of patients assessed for multimodal neuroprognostication per year was 26 (interquartile range (IQR), 22–31). Good, uncertain and poor prognoses were issued in 22%, 45.5% and 32.5% of cases, respectively. New markers have been integrated into the MMA over time, increasing from 4 to 12, as shown in Extended Data Figure 1. The median age was 53.2 (IQR, 35.7–63) years, and most of the patients were male (63.6%) and had a previously diagnosed medical condition (72%). The most common etiologies of DoC were anoxia (36.4%), TBI (18.9%) and stroke (14%). The median delay between brain injury and assessment was 33 (IQR, 23–53) days. The median time between the acquisition of the first and last marker was 6 (IQR, 2–13) days. According to expert behavioral examinations using the Coma Recovery Scale-revised (CRS-r), most of the patients (88%) were categorized as being in a minimally conscious state (MCS; 46%) or in a vegetative state/unresponsive wakefulness syndrome (VS/UWS; 42%), with few patients being comatose (3.2%) or emerging from the MCS (EMCS; 8.8%)^14^. Overall, 16.6% of patients achieved a favorable outcome, defined as GOS-E ≥ 4, after 1 year. Patients with favorable outcomes were younger, had fewer previous medical conditions and were most likely unconscious because of a TBI (37%; Table 1). Patients in a MCS had a one-year GOS-E of ≥4 more frequently than did patients in a VS/UWS (19.3% versus 3.4%), as previously reported^15,16^.

**Table 1:** Demographic and clinical characteristics of patients at time of admission for assessment

	All ICU patients, *n* = 349	GOS-E < 4; *n* = 231	GOS-E ≥ 4; *n* = 46	*P
Age in years, median (IQR)	53.2 (35.7–63)	54.6 (39.6–64.7)	38.4 (22.3–56.3)	**0.0002**
Sex (male), *n* (%)	222 (63.6)	151 (65.4)	29 (63)	0.87
Previous medical history (5 missing data)				
None, *n* (%)	97 (28.2)	57 (24.9)	19 (42.2)	**0.028**
Cardiovascular diseases, *n* (%)	172 (50)	122 (52.8)	15 (32.6)	**0.022**
Arterial hypertension, *n* (%)	113 (32.9)	84 (36.4)	9 (19.6)	**0.038**
Diabetes, *n* (%)	71 (20.6)	57 (24.7)	3 (6.5)	**0.0054**
Heart disease, *n* (%)	62 (18)	43 (18.6)	3 (6.5)	**0.050**
Psychiatric disorders, *n* (%)	80 (23.3)	63 (27.3)	8 (17.4)	0.20
Substance-use disorder, *n* (%)	47 (13.7)	38 (16.5)	3 (6.5)	0.11
Depressive disorder, *n* (%)	39 (11.3)	31 (13.4)	4 (8.7)	0.47
Neurological disease, *n* (%)	41 (11.9)	34 (14.7)	1 (2.2)	**0.015**
Stroke, *n* (%)	14 (4.1)	13 (5.6)	0	0.14
Epilepsy, *n* (%)	14 (4.1)	10 (4.3)	1 (2.2)	1.0
Cognitive dysfunction, *n* (%)	14 (4.1)	11 (4.8)	0	0.22
Cancer, *n* (%)	24 (7)	16 (6.9)	0	0.083
Etiology of DoC				
Anoxia, *n* (%)	127 (36.4)	99 (42.9)	12 (26.1)	**0.047**
Traumatic brain injury, *n* (%)	66 (18.9)	26 (11.3)	17 (37)	**6.3 × 10**^**–5**^
Stroke, *n* (%)	49 (14)	31 (13.4)	3 (6.5)	0.23
Ischemic stroke, *n* (%)	17 (4.9)	10 (4.3)	1 (2.2)	0.70
Intracerebral hemorrhage, *n* (%)	22 (6.3)	15 (6.5)	2 (4.3)	0.75
Subarachnoid hemorrhage, *n* (%)	10 (2.9)	6 (2.6)	0	0.59
Hypoglycemia, *n* (%)	15 (4.3)	13 (5.6)	0	0.14
Others, *n* (%)	66 (18.9)	39 (16.9)	11 (23.9)	0.29
CNS inflammatory disease, *n* (%)	25 (7.2)	13 (5.6)	6 (13)	0.10
CNS infectious disease, *n* (%)	6 (1.7)	3 (1.3)	1 (2.2)	0.52
Status epilepticus, *n* (%)	9 (2.6)	6 (2.6)	1 (2.2)	1.0
CNS metabolic or toxic disease, *n* (%)	6 (1.7)	5 (2.2)	0	0.59
Encephalopathy, *n* (%)	17 (4.9)	11 (4.8)	3 (6.5)	0.71
PNS disorders, *n* (%)	3 (0.9)	1 (0.4)	0	1.0
Mixed, *n* (%)	32 (9.2)	23 (10)	3 (6.5)	0.59
Brain injury–MMA delay in days, median (IQR)	33 (23–53)	29 (21–43)	35 (24–59)	0.094
Clinical state				
CRS-r, median score (IQR)	7 (5–11)	6 (4–9)	11 (8–15)	**7.5 × 10**^**–9**^
Coma, *n* (%)	11 (3.2)	10 (4.3)	0	0.38
VS/UWS, *n* (%)	146 (42)	122 (52.8)	5 (10.9)	**7.0 ×** **10**^**–8**^
MCS minus, *n* (%)	82 (23.6)	51 (22.1)	14 (30.4)	0.25
MCS plus, *n* (%)	79 (22.6)	38 (16.5)	17 (37)	**0.0037**
EMCS, *n* (%)	31 (8.9)	10 (4.3)	10 (21.7)	**0.0032**
Mechanical ventilation, *n* (%) (12 missing data)	252 (74.8)	174 (75.3)	27 (58.7)	0.084
Tracheostomy, *n* (%) (14 missing data)	136 (40.6)	84 (36.4)	23 (50)	0.091
Gastrostomy, *n* (%) (31 missing data)	45 (14.2)	30 (13)	8 (17.4)	0.33

CNS, central nervous system; PNS, peripheral nervous system. MCS patients with language related behavior (i.e. command following, intelligible verbalizations or non-functional communication) were categorised ‘MCS plus’, and otherwise ‘MCS minus’. *Two-sided Wilcoxon rank-sum test or Fisher’s exact test were used, with no adjustment for multiple comparisons. Significant differences between the two groups of patients (GOS-E of 1–3 versus 4–8) are indicated by bold *P* values (*P* < 0.05). Reported sex is sex assigned at birth.

### Disorders-of-consciousness-team prognosis predicts one-year functional outcome

**Fig. 2:**
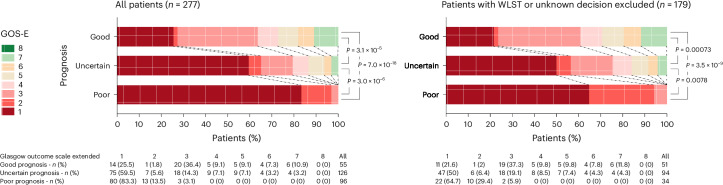
One-year functional outcome according to the DoC-team prognosis based on MMA. One-year GOS-E scores for patients with good, poor or uncertain prognoses, for all patients (left) and after exclusion of those with WLST and those for whom the WLST decision was unknown (right). The numbers of patients with each GOS-E score are shown underneath the graphs. *P* values correspond to the shift analyzes (no adjustment for multiple comparisons; see Supplementary Table 2).

The DoC-team prognosis based on MMA was significantly associated with one-year functional outcome. More precisely, a good prognosis was associated with a shift toward a better functional outcome, compared with a poor or uncertain prognosis (higher GOS-E with a common OR of 26.76 (95% confidence interval (CI), 11.88–64.39), *P* < 0.001 and 3.45 (95% CI, 1.92–6.23), *P* < 0.001; Fig. 2, left, and Supplementary Table 2). Although WLST decisions were more commonly made for those with a poor or uncertain prognosis (60.8% and 15.5%, respectively, versus 4.5% for good prognosis), the association between a good prognosis and a better functional outcome remained significant after excluding patients for whom a WSLT decision was made (n = 80) or the decision was unknown (*n* = 18; common OR, 14.57 (95% CI, 5.70–40.32) and *P* < 0.001 when compared with poor prognosis; and 2.9 (95% CI, 1.56–5.45) and *P* < 0.001 when compared with uncertain prognosis; Fig. 2, right, and Supplementary Table 2). Differences in values for each MMA marker corresponding to the DoC-team prognosis are available in Supplementary Table 1.

### Multimodal assessment improves neuroprognosis performance

**Fig. 3:**
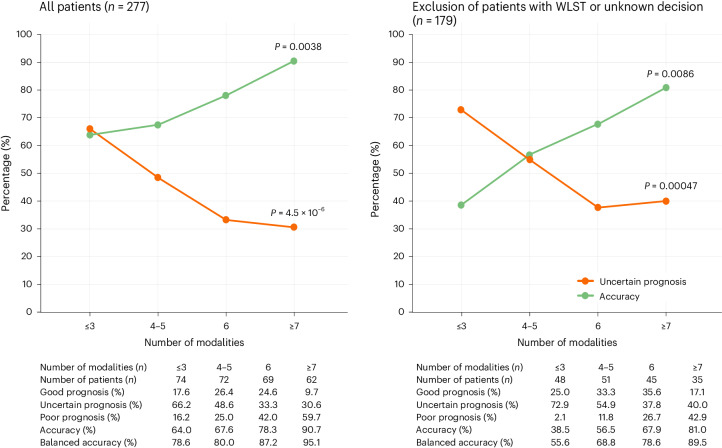
Proportion of uncertain prognoses and accuracy according to the number of modalities included in MMA. Left, accuracy and the percentage of uncertain prognoses for all patients*.* Right, accuracy and the percentage of uncertain prognoses after exclusion of those with WLST and those for whom the WLST decision was unknown*.* *P* values were calculated using Cochran–Armitage tests for trend (two-sided, no adjustment for multiple comparisons).

**Extended Data Fig. 2:**
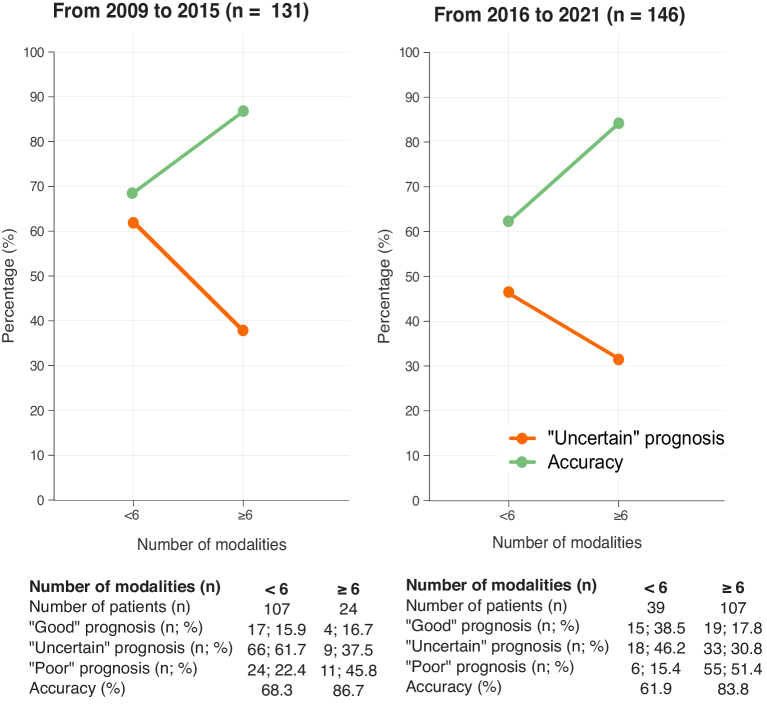
Disentangling the effect of the number of modalities and of time on accuracy. Multivariable logistic regression models with splits on medians for time ( ≥ vs < 2016) and the number of modalities ( ≥ vs < 6) suggest main effects of the number of modalities on accuracy (OR = 3.13 [95%CI: 7.29 - 7.88], *P* = 0.013, adjusted for time: OR = 0.77 [95%CI: 0.30 - 1.88], *P* = 0.568); and on “uncertain” prognosis (OR = 0.45 [95%CI: 0.25 - 0.81], *P* = 0.007, adjusted for time: OR = 0.61 [95%CI: 0.34 - 1.08], *P* = 0.088). Likelihood ratio tests did not advocate for an interaction effect of the number of modalities and time in both models (LRT, accuracy: χ2(1) = 0.003, *P* = 0.958, “uncertain” prognosis, χ2(1) = 0.307, *P* = 0.579).

As the number of modalities increases, the proportion of uncertain prognoses decreases (57.5% versus 32.1% for fewer than six modalities versus six modalities or more, respectively; OR, 0.35 (95% CI, 0.21–0.59), *P* < 0.001). In addition, the accuracy of the DoC-team prognosis improves (66.1% versus 84.3%; OR, 2.72 (95% CI, 1.18–6.47), *P* = 0.011; Fig. 3, left). The same was true when excluding patients with WLST and those for whom the WLST decision was unknown (uncertain proportion decreases from 63.6% to 38.8%, and accuracy increases from 50% to 73.5%; OR, 0.36 (95% CI, 0.19–0.69), *P* < 0.001; and OR, 2.73 (95% CI, 1.01–7.61), *P* = 0.040, respectively; Fig. 3, right). These effects remain significant when adjusted for time (Extended Data Fig. 2).

**Fig. 4:**
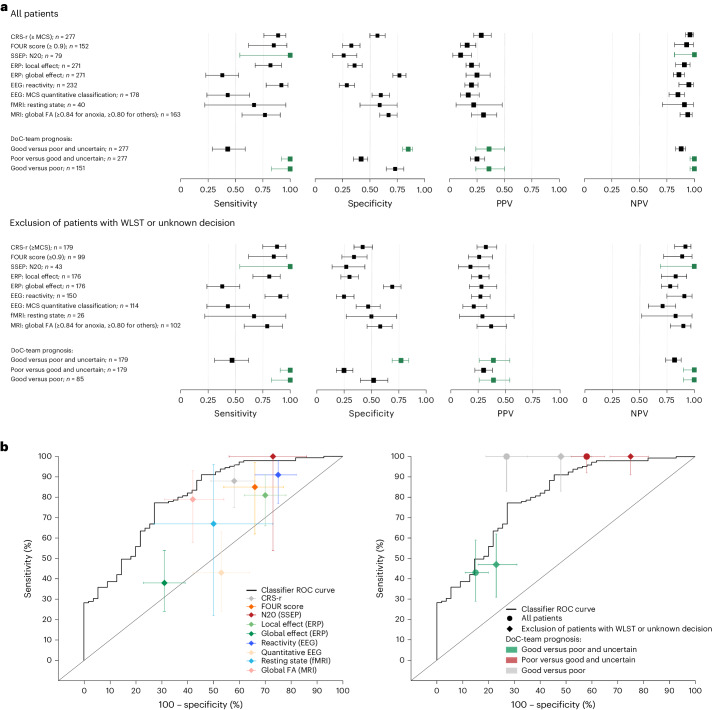
Performances of individual prognostic markers, DoC-team prognosis and multivariable classifier in predicting favorable outcomes. **a**, Performances of individual prognostic markers compared with the DoC-team prognosis performances, with all patients included (top) and after exclusion of those with WLST or for whom the WLST decision was unknown (bottom). The green color indicates the best performances (see Supplementary Table 3). Note that these figures are shown for a descriptive purpose only, because prognosis parameters have been calculated for different populations (see Supplementary Tables 4 and 5 for statistical comparisons). **b**, Performance of the multivariable classifier in predicting favorable outcomes (one-year GOS-E ≥ 4) compared with individual prognostic markers’ performances (left, training set ROC, patients with WLST or unknown decision excluded) and the DoC-team prognosis (right). See Supplementary Tables 4 and 5 for comparison of accuracy. FOUR, full outline of unresponsiveness score; FA, fractional anisotropy.

Performances of individual markers and DoC-team prognoses based on MMA (sensitivity, specificity and predictive positive and negative values) in predicting favorable outcomes are presented in Figure 4a (and Supplementary Table 3). Although some markers (for example, somatosensory evoked potential or electroencephalogram (EEG) reactivity) displayed a high sensitivity with a poor specificity, others (for example, global effect) displayed the opposite pattern. However, when compared with the DoC team’s MMA-based prognosis or the multivariable classifier (described below), no individual markers demonstrated a superior accuracy (Fig. 4b and Supplementary Tables 4 and 5).

**Extended Data Fig. 3:**
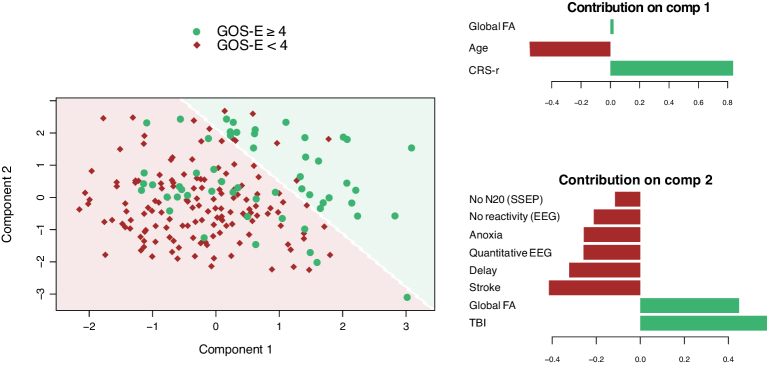
Individual plot (left) and loading plot (right) of the multivariable (sPLS-DA) classifier model using two components with respectively 3 and 8 selected prognostic markers. CRS-r: Coma Recovery Scale revised; FOUR: Full Outline of UnResponsiveness; SSEP: Somatosensory Evoked Potential; ERP: Event Related Potential; EEG: Electroencephalography; FA: Fractional Anisotropy; GOS-E: Glasgow Outcome Scale Extended.

Performance of a multivariable classifier (sparse partial least-squares discriminant analysis, see Methods) trained on MMA markers (*n* = 200 patients) was similar to that of DoC-team prognoses (accuracy of 60% versus 63.5%, respectively, see Supplementary Table 4; AUC, 0.80 on the training set; mean ± standard error cross-validated AUC, 0.73 ± 0.01; Fig. 4b, right, and Extended Data Fig. 3) and was similar to individual prognostic-marker performance (Fig. 4b, left, and Supplementary Tables 4 and 5).

## Discussion

In this study, we show for the first time that prognostication based on MMA reduces the uncertainty and increases the accuracy of prediction of long-term functional outcomes in clinically unresponsive ICU patients with brain injuries. This result strongly supports the current guidelines for neuroprognostication.

Patients with a good prognosis have a 33% chance of achieving a favorable outcome, defined by a one-year GOS-E of ≥4, corresponding to autonomy of up to 8 h per day (compared with chances of 20% and 0% for those with uncertain or poor prognosis, respectively). In the group of patients with good progneses, the proportion of those in a vegetative state (GOS-E = 2) is lower than in the uncertain or poor prognosis groups (1.8% versus 5.6% and 13.5%, respectively); however, there is an increased proportion of severe disability with dependency in this group (GOS-E = 3 for 36.4% versus 14.3% and 3.1%, respectively), a downside that will need to be addressed in future studies.

Notably, the number of modalities included in the MMA affects both uncertainty and accuracy: uncertainty decreases and accuracy increases when the number of modalities included in MMA increases. This study thus provides evidence to prompt physicians and policymakers to develop access to several neuroprognostication modalities (for example, based on EEG, event-related potential (ERP) and brain imaging), as put forth by several recent guidelines^8–10^, and not to rely on a single technique and/or otherwise, refer patients to a more specialized center^17^.

Considering the challenges of integrating MMA, it is difficult to speculate about the generalizability of our findings to other settings. However, our results primarily validate the concept that increasing the number of modalities in an assessment used by an expert team improves neuroprognostication performance. Far from being obvious, this result encourages further research into generalizing its relevance to other teams with varying levels of expertise. Although the number of possible combinations theoretically increases exponentially with the number of modalities, these modalities are not independent. Furthermore, the systematic approach that we took, including the qualitative weighting of each result in the light of the remaining evidence during the DoC-team meeting, helps mitigate this issue in most instances (for example, violation of expected hierarchical results, such as cognitive motor dissociation detection in a patient with deafness). This probably explains why uncertainty decreases with the number of modalities. Finally, although the construction of a precise decision tree was out of the scope of this work, our multivariable classifier paves the way for a more systematic approach that could be useful in optimizing the integration of MMA and reducing biases^13,18^.

Because our center is a tier-3 center within the recently proposed organization of DoC expertise in France^17^, complex cases are probably over-represented in our study. Consequently, it is possible that the performance of some individual prognostic markers could be better in a population including less complex cases. In addition, the small number of observations for some modalities could have limited the power to detect differences (for example, resting-state functional magnetic resonance imagery (fMRI) or somatosensory evoked potential (SSEP)).

The reported MMA neuroprognosis performance might be underestimated in the present study. Because our study period encompasses the last 12 years, the most recent—although validated—clinical tools that are routinely used in our center could not have been assessed properly (for example, the motor-command protocol^19^, the inextinguishable characteristic of the blink reflex^20^ and the most recent version of diffusion tensor imaging analysis assessing the whole-brain white-matter fractional anisotropy^21^). Other recent techniques independent from language abilities, such as those assessing heart–brain interaction^22,23^ and olfactory responses^24^ or transcranial magnetic stimulation with EEG^25^, could improve MMA neuroprognosis.

Because the number of modalities in the MMA increased over the study period, it is challenging to disentangle this factor from the increase in the experience and expertise of our DoC team over time (Extended Data Fig. 1). Nonetheless, accuracy did not significantly change between patients evaluated before versus after 2016, and the decrease in uncertainty seems to be better explained by the increasing number of modalities rather than the temporal factor (Extended Data Fig. 2). Finally, the fact that most of the DoC-team members (except B.R. and L.N.) have changed over the 12-year period of inclusion also advocates for the fact that the gain in performance is most likely due to the increase in the number of modalities rather than to increased experience.

Although the multivariable classifier did not perform better than the DoC team, it is worth noting that this study was not designed to compare these performances; consequently, it might lack power in that regard. However, integrating these two approaches in a new integrated MMA strategy could be promising. Indeed, analyses of the model’s weights (Extended Data Fig. 3) revealed informative features that did not appear to drive human decision-making (for example, age and delay from injury, see Supplementary Table 1). Combining objective multivariable classifier output with the specialist group’s expertise that takes into account MMA findings (as we did in this study with our DoC-team prognosis measure) might be the next step to improve prediction accuracy.

Finally, despite our effort to account for WLST, it is difficult to eliminate the self-fulfilling-prophecy bias completely. Previous studies have expressed concerns regarding patients who died following WLST but might have survived and potentially regained partial independence if life-sustaining treatments had been maintained. This classical bias can typically induce an overestimation of prognostic-test performance^26–28^. In our study, patients with a poor prognosis who eventually died after WLST were, for example, more frequently subjected to mechanical ventilation without tracheostomy and gastrostomy. Because these factors both precede the MMA and facilitate the decision for WLST, it is plausible that a bias towards a poor outcome could have been introduced^13^.

We conclude by noting that although previous studies proved the added value using multimodal approaches to go beyond pure behavioral observation and improve diagnosis in disorders of consciousness^4,8–10^, the present study extends this finding to the challenging issue of neuroprognostication.

## Methods

### Patients

We prospectively included all patients with brain injuries referred to our tertiary Neuro-ICU at La Pitié-Salpêtrière Hospital (Assistance Publique Hôpitaux de Paris - APHP Sorbonne Université, Paris, France) for expert assessment of consciousness and neuroprognosis in 2009–2021. Patients experienced sustaining consciousness disorders ranging from coma to MCS resulting from various types of brain injuries, such as anoxia, trauma, hypoglycemia, stroke or encephalitis. All reported patients were in the acute phase of their brain injury, defined by the requirement for ICU care. Patients with subacute or chronic injuries who were referred from non-ICU facilities (for example, chronic rehabilitation or nursing-home units) were not included in this study. Demographic (age, sex assigned at birth) and clinical characteristics at the time of admission for assessment were collected prospectively.

### Inclusion and ethics statement

The protocols of this observational study (NEURO-DoC/HAO-006/20130409 and M-NEURO-DoC, NCT04534777) have been preregistered and conformed to the Declaration of Helsinki and the relevant French regulations, and were approved by a local ethical committee (Ethical Committee of the French Society of Intensive Care Medicine - SRLF; Paris, France). Written informed information was delivered to and signed by a patient’s surrogate. Patients who recovered consciousness were given the opportunity to withdraw from the study.

Inclusion criteria:

1. Consciousness disorder (acute, subacute or chronic, for which our expertise was requested to better characterize the diagnosis and prognosis of recovery)
2. Brain injuries seen using computed tomography (CT) or MRI (for example, TBI, anoxia or stroke)
3. Age between 18 and 80 years

Exclusion criteria:

1. Deep sedation (for example, elevated intracranial pressure or refractory status epilepticus)
2. Severe known neurodegenerative disease (for example, Alzheimer’s disease)
3. Pregnancy

Patients admitted before 2020 were enrolled under the Neuro-DoC protocol, which served as the precursor and provided the basis for the inclusion criteria of the current protocol (NCT04534777).

The results are reported in accordance with the Strengthening the Reporting of Observational Studies in Epidemiology guidelines for reporting observational studies^29^.

### Patients’ evaluation and DoC-team meeting MMA integration

In our unit, MMA of consciousness and arrival at prognosis typically takes 1 week. During this time, patients undergo repeated behavioral assessments and electrophysiological, structural and functional brain imaging, as detailed below. As an expert tertiary center, we strive to integrate any newly published, robust and available prognosis markers into our MMA, ensuring that we use the most advanced approaches.

The synthesis of MMA takes place during an in-person meeting held by a dedicated team (the DoC team). This multidisciplinary group, comprising neurointensivists, neurologists, neurophysiologists, neuroradiologists and neuroscientists, deliberates on any discrepancies among various markers to reach a comprehensive understanding considering possible fluctuations and improvement during the week of exploration. These discussions culminate in a consensus report, which concludes with an optimistic (good), pessimistic (poor) or uncertain prognosis that is delivered to patients’ caregivers and relatives to inform decision-making on the goal of care. This final determination is hereafter referred to as the DoC-team prognosis.

During the weekly dedicated DoC-team meeting, we first present information about the patient to be discussed, reviewing their past and recent medical history; the etiology of their DoC; results from neurological examinations; behavioral scores; electrophysiological, structural or functional brain imaging; and all biological results. The meeting includes neurologists, intensivists, neuroradiologists and neurophysiologist experts in consciousness disorders.

We then systematically examine how well all these data converge across modalities to a precise outcome in the light of the GOS-E scale. Etiology has a fundamental impact in modulating the importance of structural MRI findings (that is, quantified MRI diffusion tensor imaging (DTI) of white-matter tracts) by increasing its importance, or weight, in patients with brain anoxia. Similarly, quality and how well a given result can be integrated in the light of other findings is fundamental (see red flags below). Considering the number of possible outputs and the complexity of the integration of multiple modalities, the weighting process is purely qualitative; similarly, there is no a priori universal percentage threshold for defining convergence. In any case, when a ‘reasonable convergence’ cannot be reached across exams and experts, we determine that the prognosis is uncertain.

We defined the following four items as potential red flags that decrease the value of specific exams and that can ultimately decrease our confidence and move our prognosis to the uncertain category:

- **Unusual etiology**, for which we cannot rely on substantial previous cases (for example, cerebral fat embolism or lasting doubt between anoxia and traumatic lesions in a patient with TBI with a documented resuscitated cardiac arrest).
- **Data-quality issue** (for example, movements during MRI scanning or EEG artifacts). For each of these techniques, we elaborated a data-quality assessment chart that automatically reports data quality level, facilitating fast comparison with previously published quality requirements for given modality. We systematically tried to rerun data acquisitions to correct for this limitation, when possible.
- **Strong suspicion of palsy, sensory deficit, spatial neglect or aphasia**. For instance, for patients who are suspected to be deaf, the absence of auditory-based behavioral measures or auditory cognitive ERPs is underweighted. Similarly, the absence of motor behavior in paralyzed patients is underweighted. The same logic is applied to spatial neglect and aphasia.
- **Violations of expected hierarchical** **results**. For instance, we previously documented the hierarchical structure of cognitive ERPs elicited with the local–global task. Typically, a global effect is found in individuals who also show a significant local effect and early cortical responses to sounds. Therefore, a significant global effect without a significant local effect or without univocal early cortical responses to sounds would be underweighted or even disregarded.

Finally, to optimize group decision-making and mitigate potential biases, we adhere to basic principles^13,30,31^. More specifically, to prevent the anchoring effect and promote free speech, young trainees are actively encouraged to share their opinions before more experienced group members. This approach ensures that fresh perspectives are heard first, potentially enriching the decision-making process. To circumvent the pitfall of wishful thinking, our strategy involves a clear separation between discussions focused on neuroprognostic evaluations and those pertaining to subsequent medical decisions, such as the goal of care. The latter deliberations are scheduled for separate, dedicated sessions and are informed by a broader range of factors (for example, advance directives, healthcare system limitations and the beliefs or perceptions of patients’ relatives)^13^.

### Multimodal assessment markers

#### Behavior

Behavioral assessment consisted of comprehensive neurological examinations performed by trained neurologists or neurointensivists (B.R., C.C., F.F., C.M., L.L., D.L., P.P., B.H., A.S., E.M., L.N.). In addition, for patients who were not receiving deep sedation or neuromuscular blockade, scoring was done at ICU admission, followed by each day of electrophysiology assessments using the CRS-r, a six-dimension, 23-point scale of hierarchically arranged items^32^. Patients who had been referred to our tertiary neuro-ICU for expert assessment of consciousness and prognostication receive special attention in regard to any new or unusual clinical sign noted by our staff (physicians, nurses, physiotherapists and nurse assistants). Patients’ relatives are also systematically encouraged to communicate any new and/or questionable behavior. Upon the identification of any novel sign, an additional CRS-r rating was conducted to document the patient’s current clinical state as accurately as possible. Clinical categorizations (that is coma, vegetative state (also known as VS/UWS), MCS (‘minus’ or ‘plus’) or exit from MCS) were based on the best obtained CRS-r. In addition to this expert neurological assessment and CRS-r scoring, the FOUR score was added in 2011 (ref. ^33^). In 2020, caregivers’ collective assessments of consciousness (DoC-Feeling tool) and habituation of the auditory startle reflex were added. Based on the ‘wisdom of the crowds,’ DoC-Feeling aggregates individual subjective perceptions of multiple caregivers using analog visual scales^34^. The habituation of the auditory startle reflex reflects cortical control of the blink reflex and has recently been proposed as a new sign of MCS^20^.

#### Electrophysiology

In addition to standard exploration (that is, bedside 12-electrodes spot EEG and, when indicated, SSEP), neurophysiological explorations encompassed a high-density 256-electrode EEG (hd-EEG) system (EGI) using different paradigms. All patients underwent exploration with the auditory local–global paradigm^35^. This paradigm probes different levels of cortical responses to sounds (that is, N100, mismatch negativity, P3a and P3b, reflecting cortical response, unconsciousness and conscious access to novel sounds, respectively)^36,37^. In addition to ERPs, a multivariate automatic classification of consciousness level based on the power spectrum, complexity and functional connectivity extracted from hd-EEG was implemented in 2015 (ref. ^38^) and the motor-command protocol probing brain activation in response to verbal commands (cognitive motor dissociation) on the EEG was implemented in 2021(ref. ^19^).

#### Structural and functional brain imaging

In addition to conventional structural brain imaging (standard CT or MRI scans), a quantified automatic analysis of white-matter fractional anisotropy (WM-FA) from DTI MRI was implemented in 2015(refs. ^39,40^). This method compares normalized WM-FA values measured in a large set of long-range white-matter tracts in each patient to those measured in a large cohort of patients with labeled outcomes, allowing functional outcome prediction.

Functional brain-imaging techniques, such as fMRI-resting state^41^ and [^8^F]fluorodeoxyglucose-positron emission tomography metabolic index^42,43^, assessing the preservation of the default mode network to differentiate patients in a VS/UWS and those in a MCS were implemented in 2013 and 2016, respectively.

### DoC-team prognosis and multivariable classifier predictions

As mentioned above, DoC-team prognoses based on MMA were categorized as: good (that is, when the DoC team concluded that a substantial improvement of consciousness could be expected, increasing the rationale of maintaining active and invasive life-support care), poor (that is, when the DoC team concluded that there was no evidence supporting a substantial improvement of consciousness, questioning the rationale of continuation of life support) or uncertain (that is, when the DoC team concluded that the level of uncertainty prevented confident neuroprognostication).

In addition to this DoC-team prognosis performed at the time of the MMA, we also evaluated a post hoc multivariable classifier trained on the same MMA univariate markers to predict favorable outcomes and compared performances of these two MMA-based prognoses (DoC-team prognosis by the DoC-team experts and obtained from the multivariable classifier) against each other and univariate markers (Fig. 1).

### Outcome

The primary outcome was the GOS-E (levels range from 1 to 8, with higher levels indicating better outcomes) at 12 months after the MMA. The interviewers who assessed the outcome through a structured telephone interview^44,45^ were blind to the results of the MMA conclusion. When necessary, functional outcome was dichotomized between favorable (GOS-E level ≥ 4, corresponding to a recovery of consciousness with the ability to be left up to 8 h during the day without assistance) and unfavorable (GOS-E level < 4).

Medical decisions regarding the pursuit (that is, maintaining life support and without mentioning withholding), withholding (that is, continuing ongoing therapies without escalation in case of new organ failure) or withdrawal (that is, removing or stopping ongoing therapies) of life-sustaining therapy following MMA assessment were collected. To mitigate the potential impact of the self-fulfilling prophecy, patients who underwent WLST following the MMA and those with missing information regarding the goal of care (unknown decisions) were excluded from the analysis.

### Statistics

All the statistical analyses were performed using the R software version 4.2.2 (R Development Core Team, 2022) with Rstudio version 1.4.1717.

Quantitative variables were expressed as median (with IQR) and compared using Wilcoxon rank-sum tests. Categorical variables were expressed as numbers (percentages) and analyzed using Fisher’s exact tests with OR and 95% CI, unless otherwise specified.

The association between the DoC-team prognosis and the outcome was investigated using an ordinal shift analysis, achieved by fitting a proportional odds logistic regression model to the GOS-E ordinal levels using the ‘polr’ function of the MASS R package (v7.3-58.1)^46^. The shift analysis does not require a cutoff to be determined to distinguish favorable and unfavorable outcomes, because a common OR is calculated for all cut points of the GOS-E scale, for which a value greater than one is indicative of an increased probability of a shift toward favorable outcomes (that is, higher GOS-E levels).

The performance of individual markers and DoC-team prognoses in predicting favorable outcomes was evaluated using sensitivity, specificity, positive and negative predictive values and accuracy for each test. The accuracy of the prognoses was defined as the percentage of patients with a correct prognosis (of good or poor) compared with the actual outcome. Trend analysis of uncertainty and accuracy according to the number of modalities included in the MMA was conducted using the Cochran–Armitage test^47,48^.

The multivariable classifier was developed using multivariable sparse partial least-squares discriminant analysis (sPLS-DA)^49^. This method was chosen for its ability to address both binary classification and feature-selection purposes, even in the presence of missing data in the prognostic variables. The mixOmics R package v6.22.0 (ref. ^50^) was used to determine an optimized combination of multimodal markers (derived from the models’ components as weighted sums of examination variables) by maximizing a criterion of covariance with the dichotomized GOS-E (≥4 or <4). For each component, the derived weights reflect the relative importance of the selected prognosis markers in discriminating the two groups. Missing data among the patients’ modalities were handled by the non-linear iterative partial least-squares algorithm^51^ implemented in the ‘splsda’ function^49^. Before modeling, model parameters (the number of components and the number of multimodal prognosis markers to be retained on each component) were determined using the ‘tune.splsda’ function with the balanced error rate criterion through a leave-one-out cross-validation procedure. The discriminative ability of the sPLS-DA model was assessed by a ROC analysis and two AUC values: one obtained on the training data set and the other calculated through a tenfold internal cross-validation procedure with ten repeats (mean CV-AUC ± standard error). Patients with WLST were excluded from this analysis (*n* = 200).

All statistical tests were two-sided, and the level of statistical significance was set at *P* < 0.05.

### Reporting summary

Further information on research design is available in the Nature Portfolio Reporting Summary linked to this article.

## Online content

Any methods, additional references, Nature Portfolio reporting summaries, source data, extended data, supplementary information, acknowledgements, peer review information; details of author contributions and competing interests; and statements of data and code availability are available at 10.1038/s41591-024-03019-1.

### Supplementary information

Supplementary Information Supplementary Tables 1–5.

Reporting Summary

## Data Availability

All relevant data are presented in the main manuscript, Extended Data and Supplementary Information. Additional data will be made available upon reasonable request to the corresponding author within 2 months, in compliance with the European General Data Protection Regulation.
