# Supplementary material for: Multimodal assessment improves neuroprognosis performance in clinically unresponsive critical-care patients with brain injury
Source: Nat Med. 2024 May 30;30(8):2349–55. doi: 10.1038/s41591-024-03019-1 (PMC11333287; doi:10.1038/s41591-024-03019-1)
Supplement: Supplementary file 1 — Supplementary Tables 1–5. [file 41591_2024_3019_MOESM1_ESM.pdf]

nature medicine

Supplementary information

https://doi.org/10.1038/s41591-024-03019-1

Multimodal assessment improves neuroprognosis performance in clinically unresponsive critical-care patients with brain injury

In the format provided by the authors and unedited

Supplementary information

Supplementary Table 1: Page 2-3

Supplementary Table 2: Page 4

Supplementary Table 3: Page 5

Supplementary Table 4: Page 6

Supplementary Table 5: Page 7

		DoC-team prognosis								
		**“Good” prognosis (n=69)**	**Others (n=245)**	***P**	**“Poor” prognosis (n=102)**	**Others (n=212)**	***P**	**“Uncertain” prognosis (n=143)**	**Others (n=171)**	***P**
**Age in years -** n; median (IQR)		69; 51.7 (34-62.1)	245; 52.5 (36-63.1)	0.65	102; 51.1 (37.3-63.2)	212; 52.2 (34.8-62.6)	0.72	143; 52.9 (35-63.1)	171; 51.7 (36.3-62.8)	0.97
**Previous medical history** - n (%)	No	27 (39.7)	63 (26)		22 (21.6)	68 (32.7)		41 (29.3)	49 (28.8)	
	Yes	41 (60.3)	179 (74)	**0.034**	80 (78.4)	140 (67.3)	**0.046**	99 (70.7)	121 (71.2)	1.0
**Etiology of DoC**				0.10			**3.1×10^{-8}**			**0.011**
Anoxia - n (%)		20 (29)	101 (41.2)		59 (57.8)	62 (29.3)		42 (29.4)	79 (46.2)	
TBI - n (%)		18 (26.1)	35 (14.3)		6 (5.9)	47 (22.2)		29 (20.3)	24 (14)	
Stroke - n (%)		12 (17.4)	29 (11.8)		6 (5.9)	35 (16.5)		23 (16.1)	18 (10.5)	
Hypoglycemia - n (%)		1 (1.45)	12 (4.9)		8 (7.8)	5 (2.4)		4 (2.8)	9 (5.3)	
Other - n (%)		12 (17.4)	44 (18)		11 (10.8)	45 (21.2)		33 (23.1)	23 (13.5)	
Mixed - n (%)		6 (8.7)	24 (9.8)		12 (11.8)	18 (8.5)		12 (8.4)	18 (10.5)	
**Median brain injury - MMA delay -** n; days (IQR)		69; 35 (24-64)	245; 33 (23-53)	0.55	102; 33.5 (23-51)	212; 33 (24-57.5)	0.50	143; 33 (24-56)	171; 34 (23-59)	0.89
**Median CRS-r -** n; score (IQR)		69; 12 (9-16)	245; 6 (4-9)	**1.4×10^{-14}**	102; 5 (4-6)	212; 9 (6-13)	**2.0×10^{-13}**	143; 8 (5-10)	171; 6 (5-11)	0.61
**- EMCS**	No	49 (71)	238 (97.1)		102 (100)	185 (87.3)		136 (95.1)	151 (88.3)	
	Yes	20 (29)	7 (2.9)	**1.6×10^{-9}**	0 (0)	27 (12.7)	**1.8×10^{-5}**	7 (4.9)	20 (11.7)	**0.042**
**- MCS "plus"**	No	42 (60.9)	207 (84.5)		101 (99)	148 (69.8)		106 (74.1)	143 (83.6)	
	Yes	27 (39.1)	38 (15.5)	**7.3×10^{-5}**	1 (1)	64 (30.2)	**1.4×10^{-11}**	37 (25.9)	28 (16.4)	0.050
**- MCS "minus"**	No	55 (79.7)	184 (75.1)		87 (85.3)	152 (71.7)		97 (67.8)	142 (83)	
	Yes	14 (20.3)	61 (24.9)	0.52	15 (14.7)	60 (28.3)	**0.011**	46 (32.2)	29 (17)	**0.0022**
**- VS/UWS**	No	61 (88.4)	116 (47.4)		20 (19.6)	157 (74.1)		96 (67.1)	81 (47.4)	
	Yes	8 (11.6)	129 (52.7)	**2.0×10^{-10}**	82 (80.4)	55 (25.9)	**2.9×10^{-20}**	47 (32.9)	90 (52.6)	**0.00059**
**- Coma**	No	69 (100)	235 (95.9)		98 (96.1)	206 (97.2)		137 (95.8)	167 (97.7)	
	Yes	0 (0)	10 (4.1)	0.13	4 (3.9)	6 (2.8)	0.73	6 (4.2)	4 (2.3)	0.52

Supplementary Table 1. Multimodal assessment test results according to the final DoC-team prognosis.

s

		DoC-team prognosis								
		"Good" prognosis (n=69)	Others (n=245)	*P	"Poor" prognosis (n=102)	Others (n=212)	*P	"Uncertain" prognosis (n=143)	Others (n=171)	*P
**Median FOUR score -** n; median (IQR)		31; 11 (9-13)	136; 10 (8-11)	0.064	67; 10 (8-11)	100; 10 (8-11.5)	0.76	69; 10 (8 - 11)	98; 10 (8 - 12)	0.25
**Median DoC-feeling -** n; median (IQR)		6; 56.5 (41.5-61.8)	9; 21 (12-36)	0.14	5; 12 (12-21)	10; 50 (28.5-62)	**0.032**	4; 38.3 (24.9-58.5)	11; 36 (12-56.5)	0.51
**Auditory startle reflex: habituation -** n (%)	No	0 (0)	6 (45.2)		5 (55.6)	1 (10)		1 (25)	5 (33.3)	
	Yes	6 (100)	7 (53.9)	0.11	4 (44.4)	9 (90)	0.057	3 (75)	10 (66.7)	1.0
**SSEP: N20** - n (%)	No	2 (13.5)	17 (25)		11 (27.5)	8 (18.6)		6 (21.4)	13 (23.6)	
	Yes	13 (86.7)	51 (75)	0.50	29 (72.5)	35 (81.4)	0.44	22 (78.6)	42 (76.4)	1.0
**ERP: local effect** - n (%)	No	9 (13.6)	90 (37.8)		51 (51.5)	48 (23.4)		39 (28.1)	60 (36.4)	
	Yes	57 (86.4)	148 (62.2)	**0.00017**	48 (48.5)	157 (76.6)	**$1.9x10^{-6}$**	100 (71.9)	105 (63.6)	0.14
**ERP: global effect** - n (%)	No	31 (47)	192 (80.7)		87 (87.9)	136 (66.3)		105 (75.5)	118 (71.5)	
	Yes	35 (53)	46 (19.3)	**$2.0x10^{-7}$**	12 (12.1)	69 (33.7)	**$5.0x10^{-5}$**	34 (24.5)	47 (28.5)	0.44
**EEG: reactivity** - n (%)	No	4 (7.1)	56 (27.5)		31 (34.1)	29 (17.2)		25 (22.1)	35 (23.8)	
	Yes	52 (92.9)	148 (72.6)	**0.0011**	60 (65.9)	140 (82.8)	**0.0032**	88 (77.9)	112 (76.2)	0.77
**EEG: quantitative classification** - n (%)	VS	11 (27.5)	88 (69.8)		54 (79.4)	45 (45.9)		34 (58.6)	65 (60.2)	
	MCS	29 (72.5)	38 (30.2)	**$4.9x10^{-6}$**	14 (20.6)	53 (54.1)	**$1.4x10^{-5}$**	24 (41.4)	43 (39.8)	0.87
**EEG: CMD on motor task** - n (%)	No	1 (100)	8 (100)		6 (100)	3 (100)		2 (100)	7 (100)	
	Yes	0	0		0	0		0	0	
**fMRI: resting state** - n (%)	VS	2 (28.6)	20 (58.8)		10 (62.5)	12 (48)		10 (55.6)	12 (52.2)	
	MCS	5 (71.4)	14 (41.2)	0.22	6 (37.5)	13 (52)	0.52	8 (44.4)	11 (47.8)	1.0
**MRI: median global FA -** n; median (IQR)		24; 0.89 (0.85-0.94)	102; 0.77 (0.67-0.83)	**$8.1x10^{-8}$**	62; 0.72 (0.64-0.78)	64; 0.84 (0.79-0.90)	**$9.8x10^{-8}$**	40; 0.80 (0.78-0.86)	86; 0.77 (0.68-0.88)	0.23
**PET: median metabolic index -** n; median (IQR)		2; 5.05 (4.53-5.58)	13; 3.34 (3.01-3.63)	0.062	6; 2.89 (2.72-3.30)	9; 3.63 (3.34-4.08)	**0.025**	7; 3.51 (3.30-3.86)	8; 3.2 (2.75-3.91)	0.36

Table 1 (continued).
DoC-team prognosis based on the MMA seemed to vary with the existence of previous medical history and etiologies, but not with age and with the delay from brain injury. CRS-r (but not the FOUR-score) correlated with prognosis. All markers with more than 50 observations (except for N20 on SSEP) were significantly associated with the DoC-team prognosis. DoC: Disorder of Consciousness; TBI: Traumatic Brain Injury; MMA: MultiModal Assessment; CRS-r: Coma Recovery Scale revised; (E)MCS: (Exit of) minimally conscious state; VS/UWS: Vegetative state/Unresponsive Wakefulness Syndrome; FOUR: Full Outline of UnResponsiveness Score; SSEP: Somatosensory Evoked Potential; ERP: Event Related Potential; EEG: Electroencephalography; CMD: Cognitive motor dissociation; (f)MRI: (functional) Magnetic Resonance Imaging; FA: Fractional Anisotropy; PET: Positron Emission Tomography. *Two-sided Wilcoxon sum rank test or Fisher's exact test as appropriate with no adjustment for multiple comparisons. All significant differences are indicated by the p-values in bold ($P < 0.05$).

DoC-team prognosis	All patients included			WLST patients excluded		
	**OR (95%CI)**	**Wald statistic**	***P**	**OR (95%CI)**	**Wald statistic**	***P**
**“Good” versus uncertain**	3.45 (1.92-6.23)	4.13	$3.1x10^{-5}$	2.90 (1.56-5.45)	3.34	0.00073
**“Poor” versus uncertain**	4.20 (2.26-8.16)	4.40	$3.0x10^{-6}$	2.76 (1.30-6.18)	2.57	0.0078
**“Good” versus “Poor”**	26.76 (11.88-64.39)	7.65	$7.0x10^{-18}$	14.57 (5.70-40.32)	5.39	$3.5x10^{-9}$

Supplementary Table 2.
Common odds ratios (ORs) from proportional odds logistic models.
OR: Odds ratio; CI: confidence interval; WLST: withdrawal of life-sustaining therapy; GOS-E: Glasgow Outcome Scale Extended. * Two-sided Wald chi-squared tests for odds ratio (OR) different from 1. Common ORs significantly greater than 1 indicate an increased probability of ““good”” recovery.

All patients included (n = 277)

	n	TP	TN	FP	FN	Se (95%CI)	Sp (95%CI)	PPV (95%CI)	NPV (95%CI)
CRS-r (≥ MCS "minus")	277	41	132	99	5	0.89 (0.76;0.96)	0.57 (0.50;0.64)	0.29 (0.22;0.38)	0.96 (0.92;0.99)
FOUR score (≥ 9)	152	17	43	89	3	0.85 (0.62;0.97)	0.33 (0.25;0.41)	0.16 (0.10;0.24)	0.93 (0.82;0.99)
SSEP : N20	79	6	19	54	0	1 (0.54;1)	0.26 (0.16;0.38)	0.1 (0.03;0.20)	1 (0.82;1)
ERP : local effect	271	37	82	144	8	0.82 (0.68;0.92)	0.36 (0.30;0.43)	0.20 (0.15;0.27)	0.91 (0.83;0.96)
ERP : global effect	271	17	175	51	28	0.38 (0.23;0.53)	0.77 (0.71;0.83)	0.25 (0.15;0.37)	0.86 (0.81;0.91)
EEG : reactivity	232	34	56	139	3	0.92 (0.78;0.98)	0.29 (0.22;0.36)	0.20 (0.14;0.26)	0.95 (0.86;0.99)
EEG : MCS quantitative classification	178	12	90	60	16	0.43 (0.24;0.63)	0.60 (0.52;0.68)	0.17 (0.10;0.27)	0.85 (0.77;0.91)
fMRI : resting state	40	4	20	14	2	0.67 (0.22;0.96)	0.59 (0.41;0.75)	0.22 (0.06;0.48)	0.91 (0.71;0.99)
MRI : global FA (≥ 0.84 for anoxia. ≥ 0.80 for others)	163	20	92	45	6	0.77 (0.56;0.91)	0.67 (0.59;0.75)	0.31 (0.20;0.43)	0.94 (0.87;0.98)
DoC-team prognosis : “good” versus others	277	20	196	35	26	0.43 (0.29;0.59)	0.85 (0.80;0.89)	0.36 (0.24;0.50)	0.88 (0.83;0.92)
DoC-team prognosis : “poor” versus others	277	46	96	135	0	1 (0.92;1)	0.42 (0.35;0.48)	0.25 (0.19;0.32)	1 (0.96;1)
DoC-team prognosis : “good” versus “poor”	151	20	96	35	0	1 (0.83;1)	0.73 (0.65;0.81)	0.36 (0.24;0.50)	1 (0.96;1)

All patients. WLST and unknown decision patients excluded (n = 179)

	n	TP	TN	FP	FN	Se (95%CI)	Sp (95%CI)	PPV (95%CI)	NPV (95%CI)
CRS-r (≥ MCS "minus")	179	38	57	79	5	0.88 (0.75;0.96)	0.42 (0.34;0.51)	0.32 (0.24;0.42)	0.92 (0.82;0.97)
FOUR score (≥ 9)	99	17	25	49	3	0.85 (0.62;0.97)	0.34 (0.23;0.46)	0.26 (0.16;0.38)	0.89 (0.72;0.98)
SSEP : N20	43	6	10	27	0	1 (0.54;1)	0.27 (0.14;0.44)	0.18 (0.07;0.35)	1 (0.69;1)
ERP : local effect	176	34	40	94	8	0.81 (0.66;0.91)	0.30 (0.22;0.38)	0.27 (0.19;0.35)	0.83 (0.70;0.93)
ERP : global effect	176	16	93	41	26	0.38 (0.24;0.54)	0.69 (0.61;0.77)	0.28 (0.17;0.42)	0.78 (0.70;0.85)
EEG : reactivity	150	32	29	86	3	0.91 (0.77;0.98)	0.25 (0.18;0.34)	0.27 (0.19;0.36)	0.91 (0.75;0.98)
EEG : MCS quantitative classification	114	12	40	46	16	0.43 (0.24;0.63)	0.47 (0.36;0.58)	0.21 (0.11;0.33)	0.71 (0.58;0.83)
fMRI : resting state	26	4	10	10	2	0.67 (0.22;0.96)	0.50 (0.27;0.73)	0.29 (0.08;0.58)	0.83 (0.52;0.98)
MRI : global FA (≥ 0.84 for anoxia. ≥ 0.80 for others)	102	19	45	33	5	0.79 (0.58;0.93)	0.58 (0.46;0.69)	0.37 (0.24;0.51)	0.90 (0.78;0.97)
DoC-team prognosis : “good” versus others	179	20	105	31	23	0.47 (0.31;0.62)	0.77 (0.69;0.84)	0.39 (0.26;0.54)	0.82 (0.74;0.88)
DoC-team prognosis : “poor” versus others	179	43	34	102	0	1 (0.91;1)	0.25 (0.18;0.33)	0.30 (0.22;0.38)	1 (0.90;1)
DoC-team prognosis : “good” versus “poor”	85	20	34	31	0	1 (0.83;1)	0.52 (0.40;0.65)	0.39 (0.26;0.54)	1 (0.90;1)

Supplementary Table 3.

Performances of individual prognostic markers and DoC-team prognosis to predict favorable outcome (one-year GOS-E ≥ 4). CRS-r: Coma Recovery Scale revised; MCS: Minimally conscious state; FOUR: Full Outline of UnResponsiveness; SSEP: Somatosensory Evoked Potential; ERP: Event Related Potential; EEG: Electroencephalography; (f)MRI: (functional)Magnetic Resonance Imaging; FA: Fractional Anisotropy; MMA: MultiModal Assessment; WLST: Withdraw of Life Sustaining Therapy; TP: True Positive; TN: True Negative; FP: False Positive; FN False Negative; Se: Sensibility; Sp: Specificity; PPV: Positive Predictive Value; NPV: Negative Predictive Value; CI: Confidence Interval.

Variable	N	sPLS-DA Accuracy (%)	Variable accuracy (%)	Statistic	*P	*adjusted P
**CRS-r**	200	73.5	52	24.164	$9x10^{-7}$	**$2.7x10^{-6}$**
**FOUR score**	99	72.7	46.5	14.881	0.00011	**0.00027**
**SSEP: N20**	47	76.6	40.4	10.240	0.0014	**0.0024**
**ERP: local effect**	195	73.8	42.6	34.951	$< 1x10^{-7}$	**$< 1x10^{-7}$**
**ERP: global effect**	195	73.8	62.1	6.286	0.012	**0.018**
**EEG: reactivity**	164	72.6	42.1	28.583	$1x10^{-7}$	**$4x10^{-7}$**
**EEGq: MCS classified**	122	67.2	45.9	10.417	0.0012	**0.0024**
**fMRI: resting state**	26	88.5	53.8	5.818	0.016	**0.021**
**MRI: global FA**	109	71.6	65.1	0.973	0.32	0.39
**DoC-team prognosis**	85	62.4	63.5	0.000	1	1

Supplementary Table 4.
Accuracy of the Multivariable (sPLS-DA) classifier compared to each individual markers and DoC-team prognosis (“good” vs “poor” prognosis) with *Two-sided McNemar’s chi-squared test with raw and adjusted p-values after False Discovery Rate (FDR) correction for multiple comparisons. All significant differences are indicated by the FDR-adjusted p-values in bold (FDR-adjusted $P < 0.05$). CRS-r: Coma Recovery Scale revised; SSEP: Somatosensory Evoked Potential; ERP: Event-Related Potential (‘local-global’ paradigm); EEG: Electroencephalography; MRI: Magnetic Resonance Imaging; FA: Fractional Anisotropy; sPLS-DA: sparse Partial Least Squares Discriminant Analysis.

Variable	N	DoC-team prognosis Accuracy (%)	Variable accuracy (%)	Statistic	**P*	*adjusted *P*
**CRS-r**	85	63.5	52.9	4.923	0.027	0.066
**FOUR score**	49	69.4	42.9	6.261	0.012	**0.041**
**SSEP: N20**	26	69.2	42.3	3.273	0.070	0.12
**ERP: local effect**	83	63.9	41	14.087	0.00018	**0.0018**
**ERP: global effect**	83	63.9	60.2	0.138	0.71	0.89
**EEG: reactivity**	71	67.6	43.7	10.240	0.0014	**0.0069**
**EEGq: MCS classified**	62	66.1	51.6	3.765	0.052	0.11
**fMRI: resting state**	12	75	66.7	0.000	1	1
**MRI: global FA**	53	69.8	64.2	0.364	0.55	0.78
**sPLS-DA**	85	63.5	62.4	0.000	1	1

Supplementary Table 5. Accuracy of DoC-Team's MMA-based prognosis ("good" vs "poor") compared to each individual markers and Multivariable (sPLS-DA) Classifier with *Two-sided McNemar's chi-square test with raw and adjusted p-values after False Discovery Rate (FDR) correction for multiple comparisons. All significant differences are indicated by the FDR-adjusted p-values in bold (FDR-adjusted $P < 0.05$). CRS-r: Coma Recovery Scale revised; SSEP: Somatosensory Evoked Potential; ERP: Event-Related Potential ('local-global' paradigm); EEG: Electroencephalography; MRI: Magnetic Resonance Imaging; FA: Fractional Anisotropy; sPLS-DA: sparse Partial Least Squares Discriminant Analysis.
