# Supplementary material for: Multimodal assessment improves neuroprognosis performance in clinically unresponsive critical-care patients with brain injury
Source: Nat Med. 2024 May 30;30(8):2349–55. doi: 10.1038/s41591-024-03019-1 (PMC11333287; doi:10.1038/s41591-024-03019-1)
Supplement: Supplementary file 2 — Reporting Summary [file 41591_2024_3019_MOESM2_ESM.pdf]

Corresponding author(s): Benjamin ROHAUT

Last updated by author(s): Apr 12, 2024

Reporting Summary

Nature Portfolio wishes to improve the reproducibility of the work that we publish. This form provides structure for consistency and transparency in reporting. For further information on Nature Portfolio policies, see our Editorial Policies and the Editorial Policy Checklist.

Statistics

For all statistical analyses, confirm that the following items are present in the figure legend, table legend, main text, or Methods section.

n/a	Confirmed	
☐	☒	The exact sample size (*n*) for each experimental group/condition, given as a discrete number and unit of measurement
☐	☒	A statement on whether measurements were taken from distinct samples or whether the same sample was measured repeatedly
☐	☒	The statistical test(s) used AND whether they are one- or two-sided<br>*Only common tests should be described solely by name; describe more complex techniques in the Methods section.*
☐	☒	A description of all covariates tested
☐	☒	A description of any assumptions or corrections, such as tests of normality and adjustment for multiple comparisons
☐	☒	A full description of the statistical parameters including central tendency (e.g. means) or other basic estimates (e.g. regression coefficient) AND variation (e.g. standard deviation) or associated estimates of uncertainty (e.g. confidence intervals)
☐	☒	For null hypothesis testing, the test statistic (e.g. *F*, *t*, *r*) with confidence intervals, effect sizes, degrees of freedom and *P* value noted<br>*Give P values as exact values whenever suitable.*
☒	☐	For Bayesian analysis, information on the choice of priors and Markov chain Monte Carlo settings
☒	☐	For hierarchical and complex designs, identification of the appropriate level for tests and full reporting of outcomes
☒	☐	Estimates of effect sizes (e.g. Cohen's *d*, Pearson's *r*), indicating how they were calculated

Our web collection on statistics for biologists contains articles on many of the points above.

Software and code

Policy information about availability of computer code

Data collection	No software were used to collect the data.
Data analysis	All analyses were performed using open-source software: R statistical software version 4.2.2 (R Development Core Team, 2022) with Rstudio version 1.4.1717.

For manuscripts utilizing custom algorithms or software that are central to the research but not yet described in published literature, software must be made available to editors and reviewers. We strongly encourage code deposition in a community repository (e.g. GitHub). See the Nature Portfolio guidelines for submitting code & software for further information.

Data

Policy information about availability of data

All manuscripts must include a data availability statement. This statement should provide the following information, where applicable:

- Accession codes, unique identifiers, or web links for publicly available datasets
- A description of any restrictions on data availability
- For clinical datasets or third party data, please ensure that the statement adheres to our policy

All relevant data are presented in the main manuscript, Extended Data, and Supplementary information. Additional data would be made available upon reasonable request and in compliance with the European General Data Protection Regulation to the corresponding author within 2 months.

Research involving human participants, their data, or biological material

Policy information about studies with human participants or human data. See also policy information about sex, gender (identity/presentation), and sexual orientation and race, ethnicity and racism.

Reporting on sex and gender	This research included the sex variable. Male and female sex were included as they were collected in medical reports. The number and percentages of male sex are reported in the manuscript. Of all ICU patients, 222 (63.6%) were male, and others were female (127; 37.4%).
Reporting on race, ethnicity, or other socially relevant groupings	No data regarding race, ethnicity, or other social parameters were collected in this study.
Population characteristics	349 ICU patients met our inclusion criteria. The vast majority (96%, n = 335) of the patients were referred from other ICUs for expert assessment of consciousness and neuroprognosis. DoC-team prognosis and one-year functional outcome were available for 277 (79%). The median age [IQR] was 53.2 [35.7-63] years. Most of the patients were male (63.6%) with a previous medical history (72%). The most common etiologies of DoC were anoxia (36.4%), traumatic brain injury (18.9%), and stroke (14%). The median delay between brain injury and assessment was 33 [23-53] days. The median time [IQR] between the acquisition of the first and last marker was 6 [2-13] days. According to expert behavioral examinations using the CRS-r, most of the patients (88%) were categorized as being in a Minimally Conscious State (MCS; 46%) or in a Vegetative State/ Unresponsive Wakefulness Syndrome (VS/UWS; 42%) with few comatose (3.2%) or emergent from MCS (EMCS; 8.8%) patients. Overall, 16.6% of patients achieved a favorable outcome, defined as GOS-E ≥ 4, after one year (Glasgow Outcome Scale–Extended levels range from 1 to 8, with higher levels indicating better outcomes).
Recruitment	We prospectively included all brain-injured patients referred to our tertiary Neuro-ICU at La Pitié-Salpêtrière AP-HP Sorbonne University hospital (Paris, France) for expert assessment of consciousness and neuroprognosis during the 2009–2021 period. Written informed information was delivered to patients' surrogates, and patients who recovered consciousness were given the opportunity to withdraw from the study. Since our center is a Tier-3 center within the recently proposed organization of DoC expertise in France, most complex cases are probably overrepresented in the present study.<br><br>Since our center is a Tier-3 center for DoC expertise in France, most complex cases are probably overrepresented. Consequently, it is possible that the performance of prognostic markers may be different in a population, including fewer complex cases.
Ethics oversight	The protocols of this observational study (NEURO-DoC/HAO-006/20130409 and M-NEURO-DoC/NCT04534777) conformed to the Declaration of Helsinki and French regulations and were approved by the Ethical Committee of the French Society of Intensive Care Medicine - SRLF; Paris, France.

Note that full information on the approval of the study protocol must also be provided in the manuscript.

Field-specific reporting

Please select the one below that is the best fit for your research. If you are not sure, read the appropriate sections before making your selection.

☒ Life sciences ☐ Behavioural & social sciences ☐ Ecological, evolutionary & environmental sciences

For a reference copy of the document with all sections, see nature.com/documents/nr-reporting-summary-flat.pdf

Life sciences study design

All studies must disclose on these points even when the disclosure is negative.

Sample size	As an observational prospective study, we prospectively included all brain-injured ICU patients referred to our tertiary Neuro-ICU at La Pitié-Salpêtrière AP-HP Sorbonne University Hospital (Paris, France) for expert assessment of consciousness and neuroprognosis during the 2009–2021 period. Patients suffered from sustaining disorders of consciousness related to various types of brain injury with a high degree of uncertainty questioning the goal of care. To our knowledge, our cohort represents the largest cohort of brain-injured ICU patients with neuroprognosis questioning.
Data exclusions	Patients with missing written prognosis reports and/or missing outcomes were excluded from the main analyses.
Replication	Since our study is mono-centered, a replication of our finding that increasing the number of modalities included in the multimodal assessment improves the performance of neuroprognostication (reducing uncertainty and increasing accuracy) of DoC patients is needed.
Randomization	Not applicable. This is a prospective observational study.
Blinding	The primary outcome was the ordinal score on the Glasgow Outcome Scale–Extended (GOS-E, ranging from 1 to 8, with higher levels indicating better outcomes) 12 months after the multmiodal assessment (MMA). The interviewers who performed the outcome using a structured telephone interview (as recommended) were blind to the results of the DoC-team prognosis .

Reporting for specific materials, systems and methods

We require information from authors about some types of materials, experimental systems and methods used in many studies. Here, indicate whether each material, system or method listed is relevant to your study. If you are not sure if a list item applies to your research, read the appropriate section before selecting a response.

Materials & experimental systems

n/a	Involved in the study
☒	☐ Antibodies
☒	☐ Eukaryotic cell lines
☒	☐ Palaeontology and archaeology
☒	☐ Animals and other organisms
☐	☒ Clinical data
☒	☐ Dual use research of concern
☒	☐ Plants

Methods

n/a	Involved in the study
☒	☐ ChIP-seq
☒	☐ Flow cytometry
☒	☐ MRI-based neuroimaging

Clinical data

Policy information about clinical studies

All manuscripts should comply with the ICMJE guidelines for publication of clinical research and a completed CONSORT checklist must be included with all submissions.

Clinical trial registration

NCT04534777.

Study protocol

NEURO-DoC/HAO-006/20130409 ; M-NEURO-DoC/NCT04534777.

Data collection

Data were prospectively collected in the medical neuro-ICU of La Pitié-Salpêtrière Hospital, Paris, during a 12-year period (2009-2021), including patient evaluation (multimodal assessment, MMA), DoC-team prognosis, and 1-year outcome (GOS-E) blind to the results of the DoC-team conclusion. Medical decisions regarding the goal of care (pursuit, withholding, or withdrawal of life-sustaining therapy) were collected from the medical chart.

Outcomes

The primary outcome was the ordinal score on the Glasgow Outcome Scale–Extended (GOS-E) ranging from 1 to 8, with higher levels indicating better outcomes at 12 months after the multimodal assessment (MMA). The interviewers who performed the outcome using a structured telephone interview were blind to the results of the MMA conclusion. GOS-E cutoff for dichotomized analysis was predefined as follows: favorable outcome: GOS-E ≥4, unfavorable outcome: GOS-E <4.

Plants

Seed stocks

Report on the source of all seed stocks or other plant material used. If applicable, state the seed stock centre and catalogue number. If plant specimens were collected from the field, describe the collection location, date and sampling procedures.

Novel plant genotypes

Describe the methods by which all novel plant genotypes were produced. This includes those generated by transgenic approaches, gene editing, chemical/radiation-based mutagenesis and hybridization. For transgenic lines, describe the transformation method, the number of independent lines analyzed and the generation upon which experiments were performed. For gene-edited lines, describe the editor used, the endogenous sequence targeted for editing, the targeting guide RNA sequence (if applicable) and how the editor was applied.

Authentication

Describe any authentication procedures for each seed stock used or novel genotype generated. Describe any experiments used to assess the effect of a mutation and, where applicable, how potential secondary effects (e.g. second site T-DNA insertions, mosiacism, off-target gene editing) were examined.
